# “Calcium-Phosphorus-Magnesium *Axis*” and the Metabolic Issue of Newborns Undergoing Parenteral Nutrition: Is It Time to Change Our Perspectives?

**DOI:** 10.3390/nu17050775

**Published:** 2025-02-23

**Authors:** Veronica Notarbartolo, Maurizio Carta, Bintu Ayla Badiane, Giuseppe Puccio, Giovanni Corsello, Mario Giuffrè

**Affiliations:** 1 Neonatology and Neonatal Intensive Care Unit, University Hospital “Paolo Giaccone”, 90127 Palermo, Italy; maurizio.carta@policlinico.pa.it (M.C.); gipuccio@gmail.com (G.P.); 2 Department of Health Promotion, Mother and Child Care, Internal Medicine and Medical Specialties, University of Palermo, 90127 Palermo, Italy; bintuayla.badiane@community.unipa.it (B.A.B.); giovanni.corsello@unipa.it (G.C.); mario.giuffre@unipa.it (M.G.)

**Keywords:** parenteral nutrition, preterms, metabolic complications, refeeding syndrome, newborns, calcium, phosphorus, magnesium, surgery

## Abstract

**Background:** In recent years, progress in the field of care has made prematurity an increasingly frequent phenomenon. The premature newborn, due to incompetence, is often subjected to parenteral nutrition (PN) for prolonged periods, and there may be several complications associated with it, first and foremost metabolic complications. **Methods:** In particular, the aim of this study was to evaluate how specific risk factors and/or auxological parameters influenced plasma variations in calcium, phosphorus, and magnesium levels. This is because, although little analyzed in the past, these electrolytes are essential for limiting the onset of unfavorable outcomes in neonatal age. This retrospective observational study was conducted by accessing the site intranet of the University Hospital “P. Giaccone” of Palermo, recruiting all newborns with PN necessities (106 in a total of 191), admitted to NICU in the period between 1 January 2020 and 1 January 2023. Infants undergoing PN through a central venous catheter (CVC), who remained in situ for a period ≥ 72 h, admitted to the NICU for the first time, were included. Infants with congenital malformations and/or deceased and/or transferred and/or without CVC or with CVC who remained in situ for a period < 72 h were excluded. We thus obtained 35 newborns in 2020, 33 newborns in 2021, and 38 newborns in 2022. **Results:** Hypophosphatemia was associated with a lower weight percentile (average 34.8 °C vs. 50.8 °C; *p* = 0.02) and a longer duration of PN (average 34.6 days vs. 17.3 days; *p* = 0.002). Newborns with hypercalcemia had, on average, lower gestational age (average 31.6 weeks vs. 35.7 weeks; *p* = 0.049) and weight at birth (average 1586 g vs. 2520 g; *p* = 0.038). Newborns with hypermagnesemia had, on average, higher weight and length (average weight percentile 62.1 °C vs. 42.7 °C; *p* = 0.038; average length percentile 66.7 °C vs. 44.4 °C; *p* = 0.003). Among the risk factors, cesarean section and undergoing surgery most influence the serum trend of the analyzed electrolytes. **Conclusions:** Although our results are partial and preliminary and have not always reached statistical significance, it is clear that dyselectrolytemias, in the context of metabolic complications PN-related, must be re-evaluated and carefully examined by the clinician. Prospective and controlled trials are needed to confirm our data, i.e., that the “calcium-phosphorus-magnesium axis” no longer plays only the niche role that was previously believed.

## 1. Introduction

Preterm births have increased significantly in the last 20 years, reaching incidence peaks of 11% in the two-year pandemic period 2020–2021 [1]. In Italy, extreme newborn mortality rates for preterm infants with weight < 1500 g stand at around 13.8%, compared to an international average of 15% [2]. These data are the result of an improvement in medical care offered to a category of patients “intrinsically” fragile; in this context, a correctly proportioned nutrition in micro and macronutrients, adapted to the specific needs of each newborn, plays a fundamental role [3].

Parenteral nutrition (PN) consists of the intravenous administration of carbohydrates, proteins, lipids, vitamins, and trace elements to patients who are incapable of feeding themselves; it can be total, when it is the only form of nutrition, or partial, when it is accompanied by enteral nutrition [4]. PN should be administered as soon as possible within 8–12 h of life [5,6]; in fact, its early start is crucial for a newborn’s brain development [7,8]. The method of administration of PN generally involves the use of central venous catheters (CVC) and, where possible, also peripheral venous ones (PVC) [5,9]. In the context of CVC, in the newborn, it is possible to use both the umbilical venous catheter (UVC) and the epicutaneo-caval catheter (ECC), this one inserted through the superficial veins of the limbs [10]. For many years, considerable importance has been given to mechanical, systemic, and infectious complications related to PN. In the context of metabolic complications, alterations in the carbohydrate, lipid, and amino acid profile have always played a main role [11,12]. Nevertheless, the point of view of experts has been changing since it was realized that also electrolyte disorders may be a starting point for the onset of nosological entities in newborns, especially if preterms [11,12]. Significant electrolyte abnormalities are quite rare as a direct result of PN, but the electrolyte composition of most PN solutions is suitable for maintenance needs. An inadequate intake or excessive losses can lead to deficiencies, so frequent monitoring is essential [13]. In Italy, the metabolic complications rate is 14.3% [12]. In particular, in our study, we focused on calcium-phosphorus-magnesium metabolism, which are fundamental electrolytes for bone mineralization and cellular growth [13]. Calcium, phosphorus, and magnesium imbalance is associated with several conditions such as respiratory failure, hypotension, arrhythmias, and neurological alterations [14]. Hypercalcemia is crucial for bone mineralization during fetal life but, physiologically, serum calcium level is reduced after birth [15,16], secondarily to a transient hypoparathyroidism that occurs during the first 48 h of life [17]. This condition is due to the abrupt interruption of nutrients from the placenta after birth (Placental Interrupted Feeding Syndrome or PI-Feeding Syndrome). It causes a transient catabolic status with subsequent hyperphosphatemia, hyperkalemia, and hypocalcemia [13]. According to the recent guidelines [6], at this stage, it is necessary to maintain a parenteral calcium-phosphorus intake of 1:1, also supplementing vitamin D [18,19]. The recovery of parathyroid hormone (PTH) functionality, along with an incorrect parenteral phosphorus supplementation, could be associated with hypercalcemia between the fourth and eighth day of the newborn’s life [18,20,21]. Furthermore, in this phase, a high nutritional intake based on proteins, not associated with an adequate electrolyte supplementation, can lead to the onset of a condition known as Placental Incompletely Restored Feeding Syndrome or PI-ReFeeding Syndrome (PI-RS). This depends on the fact that the “anabolic” attitude, which the organism undergoes, promotes the internalization of phosphorus and potassium and the mobilization of calcium from bone tissue, with consequent hypokalemia, hypophosphatemia, and hypercalcemia [13,22,23]. PI-RS is associated with a higher risk of developing short- and long-term complications, such as late-onset sepsis (LOS), intraventricular hemorrhage (IVH), and bronchopulmonary dysplasia (BPD) [24,25].

We present a retrospective observational study conducted on a population of 106 newborns undergoing standardized PN in a period of 3 years. The main aim of the study was to evaluate a specific metabolic complication, which concerns alterations of the “calcium-phosphorus-magnesium axis”. In particular, we evaluated a possible association between PN and the onset of changes over time in serum levels of calcium, phosphorus, and magnesium in newborns, according to specific parameters (i.e., gestational age, sex) and/or risk factors (i.e., duration of PN, undergoing surgery) analyzed. There is a twofold reason behind this study project: on the one hand, the lack of knowledge of the physiological changes that occur, first in the fetus and then in the newborn, of the hormonal *axis* of PTH; on the other hand, the little importance that has so far been given to metabolic alterations of the “calcium-phosphorus-magnesium axis” which, instead, can be at the basis of the onset of serious pathologies, especially in premature infants.

## 2. Materials and Methods

This retrospective observational study was conducted by accessing the site intranet of the University Hospital “P. Giaccone” of Palermo, recruiting all newborns with PN necessities (106 in a total of 191), and including, among others, preterms, low-weight but adeguate-for-gestational-age infants, short-statured and surgical infants, and infants needing PN as “bridging therapy”, admitted to NICU in the period between 1 January 2020 and 1 January 2023.

Inclusion criteria:

- Newborns undergoing PN through CVC remained in situ for a period ≥ 72 h.
- Newborns hospitalized at NICU for the first time.

Exclusion criteria:

- Nine newborns were excluded from the study as they were affected by congenital anomalies.
- Nine newborns were excluded as they died within the first 72 h of life.
- Thirteen newborns were excluded as the CVC remained in situ for a period < 72 h.
- Seven newborns were excluded as they were transferred on the first day of life.
- Forty-five newborns were excluded because, despite having been hospitalized in the NICU, they were not undergoing CVC procurement.
- Two newborns were excluded as they were previously discharged and hospitalized again at NICU for other reasons.

One hundred six results were obtained, divided as follows:

- 35 newborns in 2020;
- 33 newborns in 2021;
- 38 newborns in 2022.

**Figure 1:**
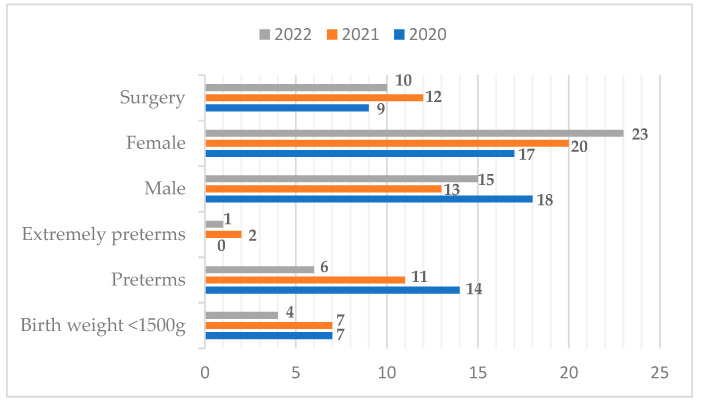
Main characteristics of the newborns studied. The numbers indicate how many infants from each subgroup have been identified.

Below is the graph showing the main characteristics of the newborns studied, divided by year (Figure 1). For extremely preterm births, we meant a gestational age < 28 weeks.

We assessed whether there was a statistical correlation between the onset of each of these complications and specific parameters:

- birth weight;
- the centile of birth weight;
- length at birth;
- the centile of length at birth;
- gestational age;
- being preterm;
- the weight class at birth;
- the gender.

We then evaluated any statistical correlation between the metabolic alterations previously listed and some risk factors:

- having undergone at least one surgical operation;
- the duration of PN;
- the average length of hospital stay.

### 2.1. Definitions

In order to guarantee homogeneity of the results, through systematic research of the more recent literature, the following definitions relating to the neonatal age were used:

- Hypophosphatemia: for serum phosphorus values ≤ 4.3 mg/dL [26].
- Hyperphosphatemia: for serum phosphorus values ≥ 9.3 mg/dL [26].
- Hypocalcemia: in the newborn with a birth weight ≥ 1500 g, a serum calcium value ≤ 8 mg/dL; in newborns with birth weight < 1500 g, a serum calcium value ≤ 7 mg/dL [27].
- Hypercalcemia: for serum calcium values ≥ 11.6 mg/dL [27].
- Hypomagnesemia: for serum magnesium values ≤ 1.5 mg/dL (also acceptable ≤ 1.34 mg/dL in the first 7 days of life) [28,29].
- Hypermagnesemia: for serum magnesium values ≥ 2.8 mg/dL (also acceptable ≥ 3.06 mg/dL in the first 7 days of life) [28,29].

### 2.2. Measurements

Each newborn was weighted, completely naked, using as digital scale a SECA^®^ brand; at the same time, using a SECA^®^ brand infantometer (SECA 416, Hamburg, Germany), the length was evaluated, understood as the maximum distance between the vertex of the head and the sole of the foot (with the child placed in a clinostatic position). Weight and length percentiles were calculated through neonatal growth curves, currently in use, IneS Charts [30]. Auxological parameters were calculated at birth in each newborn together with the respective reference centiles. Contextually, weight classes, to which newborns belong, have been calculated; in particular:

- Appropriate for gestational age (AGA) if weight >10th centile and <90th centile;
- Small for gestational age (SGA) if weight ≤10th centile;
- Large for gestational age (LGA) if weight ≥90° centile.

Gestational age was calculated by entering the last date of menstruation on the iObstetrics ^®^ app (version 3.1.6), considering any gestational age preterm <37 weeks.

In each newborn, the dosage of calcium, phosphorus, and magnesium was carried out at birth, at 48–72 h of life, at 8–10 days, and subsequently in relation to specific control needs.

The duration of PN was defined as the number of days the CVC remained in situ.

The amount of PN administered was evaluated in accordance with the nutritional intake schemes recommended in the neonatal period, currently in force [31]. In particular, starting from the birth weight and day of life of each newborn, we decided the intake of liquids to be administered and the amount of protein from which to start. The concentration of other metabolites (i.e., glucose, lipids, and electrolytes), being a standardized bag, was a direct consequence. 

Standardized PN means the use of pre-established nutrition bags (Numeta G13 and Numeta G16, emulsion for infusion, Baxter, Roma, Italy^®^), suitable for the needs of preterm and full-term newborns, respectively.

### 2.3. Statistical Analysis

The statistical analysis of the data was carried out with open-source statistical software R: R Core Team (v4.1.2; 2021). R: A language and environment for statistical computing. R Foundation for Statistical Computing, Vienna, Austria. URL: https://www.R-project.org/, accessed on 20 December 2024 [32]. Continuous variables were expressed both as mean ± standard deviation (SD) and as median and I–III quartile, as you can see from Table 1, Table 2, Table 3, Table 4, Table 5 and Table 6. The chi-square test was used to compare categorical variables independence. For comparing the distribution of a continuous variable in two or more groups, after testing the non-normality of the data, non-parametric methods (the Wilcoxon test for independent samples and Kruskal–Wallis test) have been used. For comparison between continuous variables, linear regression models were used. In particular, for longitudinal data analysis (repeated measures), linear regression mixed models were used with random intercept. A *p*-value < 0.05 was considered statistically significant [33].

## 3. Results

### 3.1. Descriptive Analysis

One hundred six newborns were enrolled; five died during hospitalization due to sepsis and three for comorbidities (heart disease and renal cystic disease). The main clinical characteristics have been summarized in Table 1 and Table 2.

**Table 1:** Main clinical characteristics of newborns who underwent PN.

Parameters	Mean (SD)	Median (25–75th Centile)
Gestational age (weeks)	35.54 ± 3.76	36.07 (32.86–38.43)
Birth weight (g)	2476.65 ± 985.37	2555 (1692.5–321.5)
Birth weight centile (°C)	45.97 ± 34.6	47 (10.5–75)
Birth length (cm)	45.26 ± 5.49	46.25 (42–49.88)
Length weight centile (°C)	44.87 ± 33.09	43 (12.5–74)
Apgar score 1′	7.01 ±2.31	8 (6–9)
Apgar score 5′	8.75 ± 1.17	9 (8–10)
Age at admission (days)	1.1 ± 3.86	0 (0–0)
Length of hospitalization (days), excluding deaths	39.28 ± 32.13	27.5 (18–52.75)
Length of hospitalization (days), including deaths	39.08 ± 31.91	27.5 (18–52.75)
Permanence of the CVC (days)	21.2 ± 23.43	13 (4–24.75)

Values have been presented as media ± standard deviation (SD) or median value (I–III quartile). CVC: central venous catheters.

**Table 2:** Main clinical characteristics of newborns who underwent PN (part 2).

Parameters	N = 106	%
Sex (M)	65	61.3
Surgery (yes)	25	23.6
Type of birth (elective CS-emergency CS-EB)	30-58-18	28.3-54.7-17
Death (yes)	8	7.5
Preterm (yes)	59	55.7
Weight classes (AGA-SGA-LGA)	64-26-16	60-25-15

Values have been presented in both numbers and percentages. CS: caesarian section; EB: eutocic birth. AGA: appropriate for gestational age. SGA: small for gestational age. LGA: large for gestational age.

During the observation period, serum phosphorus was evaluated 461 times. We reported 78 cases of hypophosphatemia and 4 cases of hyperphosphatemia.

Newborns with episodes of hypophosphatemia had, on average, lower weight (2000.6 g vs. 2596 g), although this result was not statistically significant (*p* > 0.05); moreover, they had a lower weight percentile (average 34.8 °C vs. 50.8 °C; *p* = 0.02). As easily predictable, episodes of hypophosphatemia were associated with a longer duration of PN (average 34.6 days vs. 17.3 days; *p* = 0.002) and with a longer hospital stay (57.9 days vs. 31 days; *p* = 0.00008). These results are summarized in Table 3.

**Table 3:** Characteristics comparison between newborns undergoing PN with and without hypophosphatemia.

Outcome: Hypophosphatemia		NO	YES	*p*
Gestational age (days)	Median (IQR)	36.1 (34.0 to 38.2)	36.2 (31.7 to 39.0)	0.853
Birth weight (g)	Median (IQR)	2590.0 (1802.5 to 3345.0)	2005.0 (1315.0 to 2985.0)	0.068
Birth weight centile (°C)	Median (IQR)	54.0 (14.2 to 80.8)	22.0 (3.8 to 62.2)	**0.021**
Birth length (cm)	Median (IQR)	46.8 (43.2 to 49.4)	45.0 (38.8 to 50.0)	0.270
Length weight centile (°C)	Median (IQR)	47.5 (16.0 to 75.8)	31.0 (5.8 to 65.8)	0.178
Surgery	No	60 (81.1)	21 (65.6)	0.141
	Yes	14 (18.9)	11 (34.4)	
Death	No	68 (91.9)	30 (93.8)	1.000
	Yes	6 (8.1)	2 (6.2)	
Length of hospitalization (days)	Median (IQR)	22.5 (15.0 to 42.5)	51.0 (27.2 to 63.5)	**<0.001**
Length of parenteral nutrition (days)	Median (IQR)	11.0 (4.0 to 20.0)	23.5 (10.5 to 43.0)	**0.002**

Values have been presented as median (IQR: interquartile difference).

We observed episodes of hyperphosphatemia in four patients only: each one underwent a surgical procedure. There was no association with other parameters evaluated, except the longer duration of PN (52.5 days; *p* = 0.048) and of hospital stay (71.3 days vs. 37.9 days; *p* = 0.07).

During the observation period, among 453 evaluations, we reported 27 cases of hypocalcemia and 7 cases of hypercalcemia. There was no significant statistical association between the results and the studied parameters, not even with the duration of PN or hospital stay (*p* > 0.05).

The episodes of hypercalcemia were associated with gestational age (average 31.6 weeks vs. 35.7 weeks; *p* = 0.049) and weight at birth (average 1586 g vs. 2520 g; *p* = 0.038). Although there was not statistical significance (*p* > 0.05), newborns with a hypercalcemia episode had a longer duration of PN (30.8 days vs. 22.1 days) and of hospital stay (80 days vs. 37.5 days) as well. These results are summarized in Table 4.

**Table 4:** Characteristics comparison between newborns undergoing PN with and without hypercalcemia.

Outcome: Hypercalcemia		NO	YES	*p*
Gestational age (days)	Median (IQR)	36.1 (33.6 to 38.6)	32.1 (30.1 to 32.9)	**0.048**
Birth weight (g)	Median (IQR)	2580.0 (1720.0 to 3290.0)	1450.0 (1090.0 to 1980.0)	**0.038**
Birth weight centile (°C)	Median (IQR)	46.0 (12.0 to 75.0)	58.0 (9.0 to 75.0)	0.964
Birth length (cm)	Median (IQR)	46.5 (43.0 to 50.0)	41.0 (37.0 to 42.0)	0.067
Length weight centile (°C)	Median (IQR)	42.0 (12.0 to 74.0)	46.0 (28.0 to 86.0)	0.687
Surgery	No	78 (77.2)	3 (60.0)	0.729
	Yes	23 (22.8)	2 (40.0)	
Death	No	94 (93.1)	4 (80.0)	0.832
	Yes	7 (6.9)	1 (20.0)	
Length of hospitalization (days)	Median (IQR)	27.0 (17.2 to 51.2)	48.5 (32.8 to 95.8)	0.134
Length of parenteral nutrition (days)	Median (IQR)	13.0 (4.0 to 24.0)	20.0 (19.0 to 30.0)	0.277

Values have been presented as median (IQR: interquartile difference).

During the observation period, among 352 dosages of serum magnesium, we had 3 cases of hypomagnesemia and 18 cases of hypermagnesemia. In more than half of the cases (11), the mothers of the newborns had received intravenous magnesium sulfate as prophylactic neuroprotection. Hypomagnesemia was not related to any of the parameters examined. On the contrary, newborns with hypermagnesemia had, on average, higher weight and length (average weight percentile 62.1 °C vs. 42.7 °C; *p* = 0.038; average length percentile 66.7 °C vs. 44.4 °C; *p* = 0.003). These results are summarized in Table 5.

**Table 5:** Characteristics comparison between newborns undergoing PN with and without hypermagnesemia.

Outcome: Hypermagnesemia		NO	YES	*p*
Gestational age (days)	Median (IQR)	36.1 (32.9 to 38.6)	36.2 (32.9 to 37.8)	0.451
Birth weight (g)	Median (IQR)	2550.0 (1692.5 to 3120.0)	2805.0 (1717.5 to 3530.0)	0.344
Birth weight centile (°C)	Median (IQR)	41.0 (9.0 to 72.0)	67.0 (43.2 to 96.2)	**0.037**
Birth length (cm)	Median (IQR)	46.0 (41.8 to 49.0)	47.5 (43.2 to 51.0)	0.230
Length weight centile (°C)	Median (IQR)	35.0 (9.8 to 68.0)	77.0 (52.0 to 91.8)	**0.003**
Surgery	No	69 (78.4)	12 (66.7)	0.445
	Yes	19 (21.6)	6 (33.3)	
Death	No	83 (94.3)	15 (83.3)	0.264
	Yes	5 (5.7)	3 (16.7)	
Length of hospitalization (days)	Median (IQR)	27.0 (17.0 to 49.0)	28.0 (24.0 to 59.0)	0.244
Length of parenteral nutrition (days)	Median (IQR)	13.5 (4.0 to 24.0)	12.5 (4.0 to 41.5)	0.418

Values have been presented as median (IQR: interquartile difference).

Average serum values of phosphorus, calcium, and magnesium are summarized in Table 6.

**Table 6:** Average serum values of metabolites in newborns who underwent PN.

Metabolites	Mean (SD)	Median (25–75 °C)
Phosphorus (mg/dL)	5.64 ± 1.21	5.63 (5.1–6.22)
Calcium (mg/dL)	8.92 ± 0.96	9.03 (8.46–9.54)
Magnesium (mg/dL)	2.05 ± 0.31	2 (1.8–2.23)

Values have been presented as mean standard deviation or as median (1st and 3rd quartile).

### 3.2. Longitudinal Analysis

This type of analysis was conducted by studying the time of every event in relation to the start and the end of PN. In this section, data obtained from each metabolite are analyzed separately. In particular, in the subsequent graphs, each line represents a single patient, and each point of it corresponds to a serum dosage of the examined serum metabolite. According to the longitudinal nature of the data, all the analyses were made using mixed models of regression and considering a random effect for the intercept for every single patient. The continuous red line defines the intercept of the effect fixed in time; the dashed lines under and over it, respectively, define serum cut-offs for each metabolite. On the *y*-axis we have the detected serum values, while on the *x*-axis we have the past time expressed in days [33].

#### 3.2.1. Phosphorus

**Figure 2:**
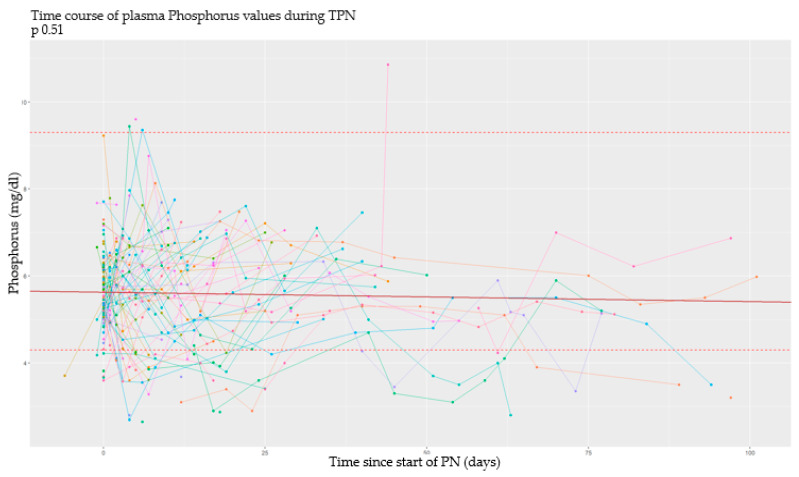
Serum phosphorus values over time.

Figure 2 shows a very mild tendency of reduction of serum phosphorus values over time that is not statistically significant (*p* > 0.05). Some newborns have this tendency in the first week of life; others have it later in the subsequent weeks.

**Figure 3:**
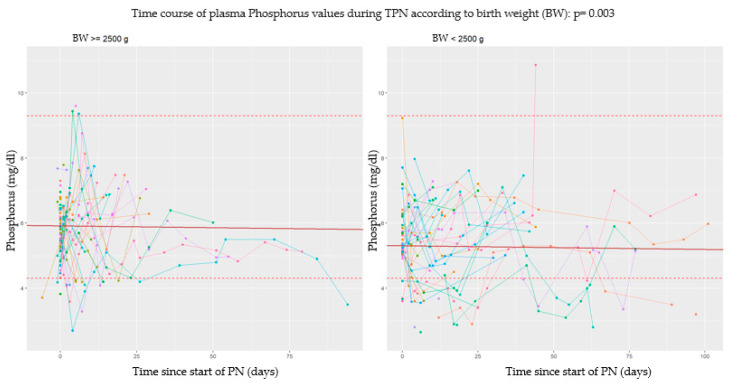
Serum phosphorus values over time: a comparison between low birth weight and normal weight newborns.

Our study showed an interesting correlation between the weight at birth and serum phosphorus level: the lower the weight at birth, the lower the serum levels of phosphorus were. This means that the effect of weight at birth on serum phosphorus level is linear. In particular, in Figure 3, we can see that normal weight infants (BW ≥ 2500 g) have on average phosphorus values 0.61 mg/dL higher compared to low weight ones.

The correlation between the tendency of serum phosphorus levels to increase with the weight percentile (*p* = 0.04) and with gestational age results statistically significant; therefore, serum phosphorus level is lower in preterm newborns compared to term ones (−0.48 mg/dL, *p* = 0.02). Another interesting result was the correlation between the length at birth (*p* = 0.0003) and length at birth percentile (*p* = 0.03) with levels of phosphatemia. In practice, a newborn with reduced length at birth has lower values of phosphatemia over time. Finally, serum phosphorus values are significantly lower (−0.70 mg/dL) in newborns born from emergency cesarean section, but not in the ones born from elective cesarean section, compared to the ones born from eutocic birth.

#### 3.2.2. Calcium

**Figure 4:**
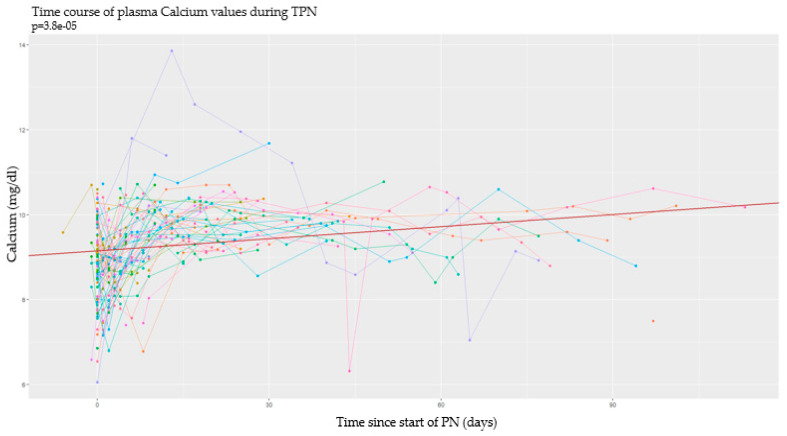
Serum calcium values over time.

During PN, calcium serum levels have the tendency to increase over time in a very significant way (Figure 4).

Neonatal anthropometric parameters are not linked with the trend of serum calcium levels. Although the correlation is weakly significant, calcemia seems to increase with low-birth-weight newborns (*p* < 0.04), in low length at birth (*p* = 0.047), in preterm infants (*p* = 0.04), and, among them, in the ones with lower gestational age (*p* = 0.03).

#### 3.2.3. Magnesium

**Figure 5:**
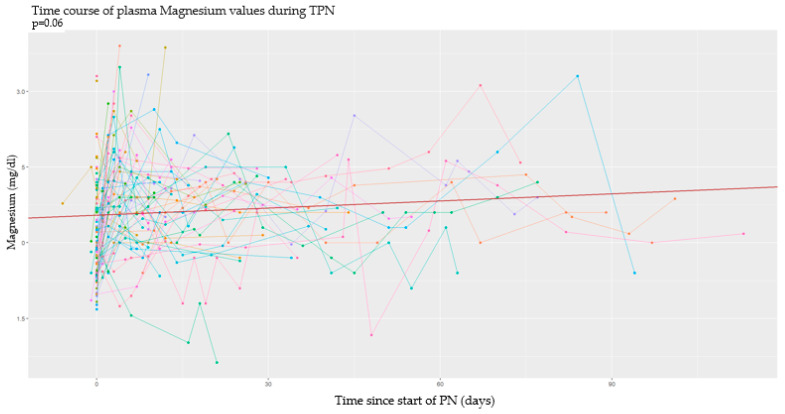
Serum magnesium values over time.

During PN, serum levels of magnesium have the tendency to increase over time, although not in a significant way (Figure 5).

The type of delivery and all the anthropometric parameters, except the length at birth (*p* = 0.048) and the length percentile (0.008), do not have a clear correlation with magnesium serum level. This means that, on average, newborns with a shorter length at birth have the tendency to have lower serum magnesium levels over time.

According to the literature data, we evaluate the modification of studied serum values over time in relation to surgical procedure, duration of PN, and average duration of hospital stay. The 23.6% of the studied cohort (*n* = 25) underwent a surgical procedure. Surgical procedure was associated with alteration of the only phosphatemia; in particular, the “surgical” newborns tend to have higher phosphatemia (+0.47 mg/dL, *p* = 0.04). Moreover, the global duration of hospital stay (excluding dead neonates) was associated with lower levels of phosphatemia (*p* = 0.02). There are no significant influence data for calcium and magnesium.

Among the deceased infants, none had statistically significant electrolyte abnormalities.

## 4. Discussion

Correct nutrition is crucial in newborns not only for their growth but also for their adequate neurocognitive development [7]. For this reason, starting an adequate and balanced PN, as soon as possible, is important when exclusive enteral feeding is not capable to meet the patient standard. A high energy intake and a low electrolytes supplementation may result in PI-Refeeding Syndrome, which manifests itself with hypophosphatemia and hypercalcemia in newborns [13,14,22,34]. The use of standardized PN limits this condition, allowing the newborn to be given quantities of macro- and micronutrients that are adequate to his needs, with nutritional objectives to be achieved in a more balanced way, based on birth weight and day of life [35,36]. Nevertheless, as our study highlights, even the use of standardized PN can lead to the more or less early onset of dyselectrolytemia.

In our study we evaluated a possible association between PN and the onset of changes over time in serum levels of calcium, phosphorus and magnesium in newborns, according to specific parameters (i.e., gestational age, sex) and/or risk factors (i.e., duration of PN, undergoing surgery) analyzed.

Although the reduction of blood levels of phosphorus does not reach statistical significance in newborns undergoing PN, it is evident that a lower birth weight and being preterm are strongly correlated to a reduction in phosphorus levels over time (*p* < 0.05), with a simultaneous increase in calcemia levels. These results, as already mentioned epiphenomenon of the PI-Refeeding Syndrome, are in agreement with the data in the recent literature [13,22,36]. At the same time, analyzing thoroughly single episodes of hypophosphatemia and hypercalcemia, these alterations resulted more frequent in premature and in low-birth weight neonates. Zhang et al. [37] demonstrated that male newborns had the tendency to have lower phosphatemia compared to female ones, due to major renal immaturity and subsequently a stronger phosphaturic effect. We did not report the same result (probably due to the small sample size), so further studies are necessary to evaluate the influence of sex in the onset of PN-related metabolic complications. Neonates born from emergency cesarean section, but not the ones born from elective cesarean section, have lower phosphatemia compared to the ones born from eutocic delivery. There are no conclusive results regarding physiopathological explanation, but this event is likely due to a less physiological passage of phosphorus from the mother to the fetus, as stated in previous works [15,16]. In PI-Refeeding Syndrome it is also possible to have hypomagnesemia, although the etiological mechanism is yet unknown [14]. In 2016 Mehta et al., highlighted that serum magnesium levels are highly influenced by birth weight, prematurity and weight class of the neonate [38]. Nevertheless, in our study, serum magnesium values had the tendency to increase during PN, although not statistically significant.

Our results show how “calcium-phosphorus-magnesium axis” plays a key role in the metabolic structure of the newborn, especially if preterm and how, in this context, a correct PN is fundamental to avoid gross imbalances. The finding of dyselectrolytemia must be a warning for the implementation of changes in the management of PN, as was performed by us when the electrolytic alteration was highlighted. A correct and balanced electrolyte supplementation, in accordance with the nutritional schemes currently in force in the neonatal sector [31], is very important to guarantee the correct maintenance of calcium *x* phosphorus product, to avoid bone mineralization deficit, very common in neonates. The early discovery of electrolyte alterations involves the modification of nutritional patterns: although our study did not correlate metabolic and clinical outcomes, and although it was not specified how the clinician acted upon the discovery of dyselectrolytemia (this being outside the scheme of study), it is known how metabolic imbalance may predispose to an unhealthy outcome [20,24,25].

An important growth happens in the third trimester of pregnancy, mostly due to a high number of electrolytes that are carried from the placenta to the fetus [13,15]. Without a correct intake of electrolytes, the neonate can develop a severe osteopenia that in the future can be associated with short stature [39]. For this reason, in our study we evaluated an eventual correlation between the trend of serum calcium, phosphorus, and magnesium values and the birth length. Our results have highlighted that newborns with low length at birth tend to have lower phosphorus and magnesium levels over time, in front of higher calcium levels. This correlation is not well described in the literature [40,41]. Contextually, in our sample, hypermagnesemia was reported in higher birth weight and longer neonates that had higher electrolyte reserves. It is well known that a low calcium, magnesium, and phosphorus intake during pregnancy can cause a decreased bone growth resulting in a lower birth length [41,42]. Therefore, it is clear that phosphorus and magnesium’s tendency to decrease over time in lower birth length newborns may be due to poor supply [13,15,23]. It is less clear the association between low birth length and serum calcium tendency to increase [34,43,44,45].

Gastrointestinal surgical procedures are associated with an increase in phosphatemia values over time and with a higher number of episodes of hyperphosphatemia; these results seem to contradict the literature data in which neonates who have undergone abdominal surgery have generally lower phosphatemia values due to its altered reabsorption [46,47,48]. This discrepancy is probably due to the fact that in our cohort, 56% of surgical neonates did not have procedures in the small intestine where phosphorus reabsorption mainly occurs. Among the other factors examined, it was found that a longer duration of PN and days of hospitalization are correlated with a higher rate of dyselectrolytemia, especially with regards to phosphorus. This result needs further studies. Although the small number of the sample does not allow us to create a standardized procedure that establishes a correct dosage timing of the analyzed electrolytes, our study underlines and confirms the importance of their close monitoring to prevent consequences on the health of newborns.

### Limitations of the Study

The main limits of our study were the small number of patients, a partially homogeneous sample, and the retrospective analysis, as well as the lack of real standardization of the timing of analyses carried out in the enrolled newborns. The poor percentage of intrauterine growth restriction (7%) in our cohort did not allow its correlation with electrolyte alterations, in particular with phosphatemia, a well-known association in the current literature [49,50,51].

At the same time, longitudinal analysis of data had low accuracy; therefore, prospective and controlled studies are needed to give indications on possible associations between low and high blood levels of electrolytes and risk factors. The absent comparison between partial parenteral nutrition and total parenteral nutrition and the lack of correlation between electrolyte alterations and specific clinical manifestations in affected newborns (e.g., osteopenia of prematurity) are other limits of the study.

## 5. Conclusions

Although our results are partial and preliminary and have not always reached statistical significance, it is clear that dyselectrolytemias, in the context of metabolic complications PN-related, must be re-evaluated and carefully examined by the clinician. To date, limitations of our study do not allow us to reach conclusive data, and further studies are certainly necessary. What our study allows us to affirm is that the metabolic axis we examined is certainly associated with specific clinical parameters and specific risk factors. The serial, daily control of electrolytes in newborns undergoing PN is, therefore, fundamental in order to intervene as early as possible, starting from the assumption that the “calcium- phosphorus-magnesium axis” no longer plays only the niche role that was previously believed, but its modulation is essential to prevent the onset of future endocrine disorders in children.

## Data Availability

The original contributions presented in the study are included in the article, further inquiries can be directed to the corresponding author due to privacy.

## References

[1] www.sin-neonatologia.it.

[2] www.iss.it.

[3] Calkins K.L., Venick R.S., Devaskar S.U. (2014). Complications associated with parenteral nutrition in the neonate. Clin. Perinatol..

[4] Hamdan M., Puckett Y. (2022). Total Parenteral Nutrition.

[5] NICE (2020). Neonatal Parenteral Nutrition. NICE Guideline.

[6] Bolisetty S. (2022). Newborn Parenteral Nutrition. NSW Guideline. https://www.seslhd.health.nsw.gov.au/.

[7] Waffa E.M.A.M., Islahudin F., Shamsuddin A.F. (2014). Complications of Parenteral Nutrition in Neonates. Res. J. Pharm. Tech..

[8] Velaphi S. (2011). Nutritional requirements and parenteral nutrition in preterm infants. S Afr. J. Clin. Nutr..

[9] Embleton N.D., Simmer K. (2014). Practice of Parenteral Nutrition in VLBW and ELBW Infants. Nutr. Care Preterm Infants.

[10] Grover T.R., Weems M.F., Brozanski B., Daniel J., Haberman B., Rintoul N., Walden A., Hedrick A., Mahmood B., Seabrook R. (2022). Central Line Utilization and Complications in Infants with Congenital Diaphragmatic Hernia. Am. J. Perinatol..

[11] Patel P., Bhatia J. (2016). Total parenteral nutrition for the very low birth weight infant. Semin. Fetal Neonatal Med..

[12] Mantegazza C., Landy N., Zuccotti G.V., Köglmeier (2018). Indications and complications of inpatient parenteral nutrition prescribed to children in a large tertiary referral hospital. Ital. J. Pediatr..

[13] Bonsante F., Iacobelli S., Latorre G., Rigo J., De Felice C., Robillard P.Y., Gouyon J.B. (2013). Initial Amino Acid Intake Influences Phosphorus and Calcium Homeostasis in Preterm Infants—It Is Time to Change the Composition of the Early Parenteral Nutrition. PLoS ONE.

[14] Da Silva J.S.V., Seres D.S., Sabino K., Adams S.C., Berdahl G.J., Citty S.W., Cober M.P., Evans D.C., Greaves J.R., Gura K.M. (2020). ASPEN Consensus Recommendations for Refeeding Syndrome. Nutr. Clin. Pract..

[15] Kovacs C.S. (2015). Calcium, phosphorus, and bone metabolism in the fetus and newborn. Early Hum. Dev..

[16] Vuralli D. (2019). Clinical Approach to Hypocalcemia in Newborn Period and Infancy: Who Should Be Treated?. Int. J. Pediatr..

[17] Mihatsch W., Fewtrell M., Goulet O., Molgaard C., Picaud J.-C., Senterre T., ESPGHAN/ESPEN/ESPR/CSPEN working group on pediatric parenteral nutrition (2018). ESPGHAN/ESPEN/ESPR/CSPEN guidelines on pediatric parenteral nutrition: Calcium, phosphorus and magnesium. Clin. Nutr..

[18] Christmann V., de Grauw A.M., Visser R., Matthijsse R.P., van Goudoever J.B., van Heijst A.F.J. (2014). Early Postnatal Calcium and Phosphorus Metabolism in Preterm Infants. J. Pediatr. Gastroenterol. Nutr..

[19] Johnson P.J. (2014). Review of Micronutrients in Parenteral Nutrition for the NICU Population. Neonatal Netw..

[20] Moscatelli A., Buratti S., Buffoni I. (2022). Raccomandazioni per la Nutrizione Parenterale in Terapia Intensiva Neonatale e Pediatrica. https://www.gaslini.org/reparti/terapia-intensiva-neonatale-e-pediatrica.

[21] Tinnion R.J., Embleton N.D. (2012). How to use… alkaline phosphatase in neonatology. Arch. Dis. Child. Educ. Pract..

[22] Brener Dik P.H., Galletti M.F., Bacigalupo L.T., Fernández Jonusas S., Mariani G.L. (2018). Hypercalcemia and hypophosphatemia among preterm infants receiving aggressive parenteral nutrition. Arch. Argent. Pediatr..

[23] Griffin I.J. (2022). Parenteral Nutrition in Premature Infants.

[24] Mizumoto H., Mikami M., Oda H., Hata D. (2021). Refeeding syndrome in a small-for-dates micro-preemie receiving early parenteral nutrition. Pediatr. Int..

[25] Moltu S.J., Strømmen K., Blakstad E.W., Almaas A.N., Westerberg A.C., Braekke K., Rønnestad A., Nakstad B., Berg J.P., Veierød M.B. (2013). Enhanced feeding in very-low-birthweight infants may cause electrolyte disturbances and septicemia—A randomized, controlled trial. Clin. Nutr..

[26] www.medscape.org.

[27] Abrams S.A. (2022). Neonatal Hypocalcemia.

[28] Fanaroff A.A., Fanaroff J.M. (2020). Cure del Neonato ad Alto Rischio.

[29] Rigo J., Pieltain C., Christmann V., Bonsante F., Moltu S.J., Iacobelli S., Marret S. (2017). Serum Magnesium Levels in Preterm Infants Are Higher Than Adult Levels: A Systematic Literature Review and Meta-Analysis. Nutrients.

[30] www.inescharts.com.

[31] Gruppo di Nutrizione Parenterale Neonatale (2017). Manuale di Nutrizione Parenterale Neonatale.

[32] www.R-project.org/.

[33] Triola M.M., Triola M.F., Giraudo M.T. (2022). Fondamenti di Statistica Biomedica. Per le Discipline Biomediche.

[34] Cormack B.E., Harding J.E., Miller S.P., Bloomfield F.H. (2019). The Influence of Early Nutrition on Brain Growth and Neurodevelopment in Extremely Preterm Babies: A Narrative Review. Nutrients.

[35] Al-Wassia H., Lyon A.W., Rose S.M., Sauve R.S., Fenton T.R. (2019). Hypophosphatemia is Prevalent among Preterm Infants Less than 1,500 Grams. Am. J. Perinatol..

[36] Igarashi A., Okuno T., Ohta G., Tokuriki S., Ohshima Y. (2017). Risk Factors for the Development of Refeeding Syndrome-Like Hypophosphatemia in Very Low Birth Weight Infants. Dis. Markers.

[37] Zhang H., Jia Q., Piao M., Chang Y., Zhang J., Tong X., Han T. (2021). Screening of Serum Alkaline Phosphatase and Phosphate Helps Early Detection of Metabolic Bone Disease in Extremely Low Birth Weight Infants. Front. Pediatr..

[38] Mehta Y., Shitole C., Setia M.S. (2016). Factors Associated With Changes in Magnesium Levels in Asymptomatic Neonates: A Longitudinal Analysis. Iran. J. Pediatr..

[39] Pereira-da-Silva L., Macedo I., Rosa M.L., Bridges K.M. (2014). Calcium and Phosphorus Intake by Parenteral Nutrition in Preterm Infants. Diet and Nutrition in Critical Care.

[40] Colak A., Yildiz O., Toprak B., Turkon H., Halicioglu O., Coker I. (2016). Correlation between calcium and phosphorus in cord blood and birth size in term infants. Minerva Pediatr..

[41] Doi M., Rekha R.S., Ahmed S., Okada M., Roy A.K., El Arifeen S., Ekström E.C., Raqib R., Wagatsuma Y. (2011). Association between calcium in cord blood and newborn size in Bangladesh. Br. J. Nutr..

[42] Improda N., Mazzeo F., Rossi A., Rossi C., Improda F.P., Izzo A. (2021). Severe hypercalcemia associated with hypophosphatemia in very premature infants: A case report. Ital. J. Pediatr..

[43] Kovacs C.S., Ward L.M. (2020). Physiology of Calcium, Phosphorus, and Bone Metabolism During Fetal and Neonatal Development. Maternal-Fetal and Neonatal Endocrinology.

[44] Pająk A., Królak-Olejnik B., Szafrańska A. (2018). Early hypophosphatemia in very low birth weight preterm infants. Adv. Clin. Exp. Med..

[45] Guthrie G., Burrin D. (2021). Impact of Parenteral Lipid Emulsion Components on Cholestatic Liver Disease in Neonates. Nutrients.

[46] Dokos C., Tsakalidis C., Manaridou K., Karayianni P., Kyrkos I., Roussos I. (2017). Clinical-laboratory findings of bone metabolism in healthy premature and full-term neonates: Preliminary results. Clin. Cases Miner. Bone Metab..

[47] Chhavi N., Ojha S., Awasthi A., Shalimar, Goel A. (2022). Serum Level of Alanine and Aspartate Aminotransferase Levels in Newborns in India. J. Clin. Exp. Hepatol..

[48] Gatti S., Quattrini S., Palpacelli A., Catassi G.N., Lionetti M.E., Catassi C. (2022). Metabolic Bone Disease in Children with Intestinal Failure and Long-Term Parenteral Nutrition: A Systematic Review. Nutrients.

[49] Davidson J., Tong S., Hauck A., Lawson D.S., Jaggers J., Kaufman J., da Cruz E. (2012). Alkaline phosphatase activity after cardiothoracic surgery in infants and correlation with post-operative support and inflammation: A prospective cohort study. Crit. Care.

[50] Bradford C.V., Cober M.P., Miller J.L. (2021). Refeeding Syndrome in the Neonatal Intensive Care Unit. J. Pediatr. Pharmacol. Ther..

[51] Mihatsch W., Thome U., de Pipaon M.S. (2021). Update on Calcium and Phosphorus Requirements of Preterm Infants and Recommendations for Enteral Mineral Intake. Nutrients.

